# Synthesis, characterization and application of organoclays for adsorptive desulfurization of fuel oil

**DOI:** 10.1038/s41598-022-11054-6

**Published:** 2022-05-05

**Authors:** Muhammad Saeed, Aqsa Riaz, Azeem Intisar, Mazhar Iqbal Zafar, Humaria Fatima, Haidar Howari, Aiyeshah Alhodaib, Amir Waseem

**Affiliations:** 1 School of Chemistry, University of the Punjab, Lahore, 54590 Pakistan; 2 Department of Environmental Sciences, Faculty of Biological Sciences, Quaid-i-Azam University, Islamabad, 45320 Pakistan; 3 Department of Pharmacy, Faculty of Biological Sciences, Quaid-i-Azam University, Islamabad, 45320 Pakistan; 4 Department of Physics, Deanship of Educational Services, Qassim University, Buraydah, 51452 Saudi Arabia; 5 Department of Physics, College of Science, Qassim University, Buraydah, 51452 Saudi Arabia; 6 Department of Chemistry, Quaid-i-Azam University, Islamabad, 45320 Pakistan

**Keywords:** Green chemistry, Materials chemistry, Fossil fuels

## Abstract

The present study encompasses the application of cost effective, organo-modified bentonite material for efficient desulfurization of model oil and real fuel. For the adsorptive desulfurization of oil, dibenzothiophene (DBT) was used as model compound. Various experimental parameters (time, temperature, adsorbent-amount and DBT concentration) were thoroughly investigated. The synthesized material was characterized via X-ray diffraction (XRD), X-ray Fluorescence (XRF), Scanning electron microscopy (SEM), Energy dispersive x-ray (EDX), Thermogravimetric analysis (TGA) and Fourier transform infrared spectroscopy (FT-IR). The modification exhibits the increase in interlayer spacing of clay as confirmed from XRD and modified material shows interesting morphology as compared to unmodified bentonite. The results showed that > 90% of DBT removal was achieved under optimized conditions for B-BTC, B-BTB and B-DSS and > 80% for B-BEHA, for model fuel oil which are greater than unmodified clay (< 45%). Additionally, the findings from desulfurization of real fuel oil declare that 96.76% and 95.83% removal efficiency was achieved for kerosene and diesel oil respectively, at optimized conditions and fuel properties follow ASTM specifications. The obtained findings well fitted with thermodynamic, isothermal (Langmuir) with adsorption capacity (70.8 (B-BTC), 66 (B-BTB), 61.2 (B-DSS) and 55.2 (B-BEHA) in mg/g) and pseudo-second-order kinetics. In thermodynamic studies, negative sign ($\Delta G^\circ )$ specifies the spontaneity whereas, $\left(\Delta H^\circ \right)$ endothermic and positive sign $(\Delta S^\circ )$ show randomness after DBT adsorption onto organoclay.

## Introduction

Demand for the utilization of more eco-friendly fuels and their production is increasing due to the implementation of legislation requiring strict regulation of green-house gas emissions. Many countries are currently enforcing a strict control of sulfur content in liquid fuels to ultralow levels, making the production of deep desulfurization processes an important research objective^1–3^. Among the main industrial processes for the removal of sulfur from liquid fuels, the most important is referred as adsorptive desulfurization (ADS) and operates with microporous and mesoporous materials^4–6^.

As the stringent sulfur content has emerged, desulfurization of fuel has received massive attention of the world. Thiols, sulfides and disulfides can be efficiently removed using conventional adsorbents^7^. Various important features of adsorptive desulfurization (ADS) which have grasped attention as an alternative technology are high efficiency, moderate operation conditions and its economical rates. Similar technique is photocatalytic oxidative desulfurization (ODS) in which sulfur compounds are converted to SO_4_^–2^, sulfoxides and sulfones under photo-oxidation^8^. These polarized compounds can be separated from non-polar oils into extractants i.e., water, acetonitrile or ionic liquids (ILs). Numerous studies have been conducted for desulfurization of fuel oils by combining UV irradiation and liquid–liquid extraction. The center of their research (adsorptive and oxidative) lies on product identification and the effect of sulfur removal^9^.

These days many of the refineries utilize hydrodesulfurization method (HDS) for the removal of sulfur compounds from petroleum products. The process contains drastic conditions as it operates at high temperatures, elevated concentrations of hydrogen gas as well as not sufficient for the conversion of aromatic sulfur compounds (thiophene, benzothiophene, dibenzothiophene and their derivatives) into corresponding sulfoxides and sulfones efficiently. Despite its severe conditions the process is unable to achieve current sulfur specifications^9–11^. Due to this reason the researchers are working to develop alternate desulfurization methods. Many methods are reported for sulfur removal such as oxidative desulfurization (ODS), bio-desulfurization (BDS) and adsorptive desulfurization (ADS)^8^. Desulfurization assisted by adsorption a potential method to remove sulfur compounds from liquid fuels. During the last few decades, adsorptive removal has gained considerable research interests upon modification that result in increased the adsorption capacity. Based on experimental results, about 99% of the sulfur compounds can be removed from model diesel fuels to reach promising desulfurization via adsorption process^12,13^. Adsorption is considered to be one of most popular technique for sulfur removal owing to its high efficacy, cost efficiency, simple operation, and tolerant of processing conditions^14^. Commonly used adsorbents are grapheme nanoplates^15^, mesoporous silica^16^, magnetic carbon^17^, activated alumina^18^, activated charcoal^19^, Tin (Sn) impregnated activated charcoal^20^, activated carbon manganese oxide nanocomposite^21^, mesoporous carbon^22^, activated carbon^23^, etc. but they exhibit lower adsorption capacity as compared to clay material.

Clay (low-cost and eco-friendly) is a naturally available adsorbent that has been used for the removal of dyes, heavy metals, organic pollutants, mycotoxins, sulfur content etc. form decades^24–26^. Its effectiveness is because of its layered structure that bears strong affinity regarding cations and anions and has exchangeable ions that play vital role in adsorption. Thus, it works as host material for the adsorbents with an increased surface area as well as it has significant applications in synthesis of biodiesel^27,28^. However, the adsorption capacity of raw clay is not as good as compared to synthetically modified clay. Once the clay surface is modified with surfactant based organic molecule, its adsorption capacity could be enhanced by leaps and bounds. For the past two decades removal of organic contaminants using modified solid material has gained much attention^29,30^. The clay was modified with organic molecules to form micelle-like structures on its surface that had the capability to solubilize organic pollutant such as DBT.

We herein report the removal of DBT desulfurization by a low-cost adsorbent i.e., organoclay. But hydrophilicity of interlayer surface restricts the adsorption capacity of clay for organic targets. So, benzyl tri-n-butyl ammonium bromide (BTB), Dioctyl sodium sulfosuccinate (DSS), benzethonium chloride (BTC) and Bis (2-ethylhexyl) amine (BEHA) based modified clay was observed to have improved hydrophobicity to interact strongly with the organic matter providing efficient adsorption and first time used for the removal of DBT from model fuel oil and real fuel oil. In addition, the unmodified and modified clay was characterized via FT-IR, XRF, XRD, SEM, EDX and TGA to investigate the composition of material. Adsorption studies reveal the desulfurization of mg/L level of DBT in model fuel and real fuel oil. To further investigate the adsorption capacity of the clay, adsorption kinetics (pseudo first order, pseudo second order and intraparticle diffusion model) and isotherms (Langmuir, Freundlich and Temkin model) were also studied and efficacy was monitored at different optimized conditions.

## Materials and methods

### Materials

Sodium Bentonite (Al_2_H_2_Na_2_O_13_Si_4_, LOT: 10195902) pristine clay (2:1) (BT) and modification reagent; benzyl tri-n-butyl ammonium bromide (BTB), Dioctyl sodium sulfosuccinate (DSS), benzethonium chloride (BTC) and Bis (2-ethylhexyl) amine (BEHA) were obtained from *Alfa Aesar Co.,* Germany and used without any modification. The targeted adsorbate Dibenzothiophene (DBT) was obtained from *Sigma Aldrich Co.,* used for adsorption study.

### Material synthesis

Modified bentonite (B-BTB, B-DSS, B-BTC and B-BEHA) were synthesized via modification regent by dispersing the 1 g of bentonite (BT) in 50 mL of water through sonication (for the dispersion of clay particles into water) for 30 min at room temperature, reported previously^24–26^. The pH (4) was maintained by using 0.5 M HCl to make clear suspension of above solution. 0.3 g of modification reagent (BTB, DSS, BTC or BEHA) was dissolved in 50 mL of water and added into above suspension and refluxed stirring was carried out for 6 h at 120 °C. The synthesized material was subjected to filtration, dried at 120 °C in oven and grounds via pestle mortar.

### Adsorption studies method

Adsorption of Dibenzothiophene (DBT) on to unmodified clay (BT) and synthesized organoclay (B-BTB, B-DSS, B-BTC and B-BEHA) was conducted with different concentrations of DBT in isooctane. The optimization studies were performed with 30 mL of 1000 mg/L DBT solution with 0.5 g of organoclay. Centrifugation and filtration were used for the separation of adsorbent and adsorbate (DBT solution). The calibration curve was obtained by UV/Vis spectrophotometer (Shimadzu UV1700 Japan) and PETRA X-Ray Sulfur Analyzer, ASTM D-4294) was used to analyze the residual amount of DBT. The percentage efficiency (S%) and adsorption capacity (q_e_) at equilibrium for adsorption of DBT were determined by following Eqs. (1) and (2)^24,31^:

1 $$ Sorption\, Efficiency\, \left( {S\% } \right) = \frac{{\left( {Ci - Ce} \right)}}{Ci} \times 100 $$

2 $$ qe = \frac{{\left( {Ci - Ce} \right) \times V}}{m} $$

Here, C_e_ and C_i_ = equilibrium and initial DBT concentrations (mg/L), qe = adsorption capacity of synthesized organoclay (mg/g), V = solution volume, m = mass of organoclay by weight (g).

### Instrumentation

The unmodified bentonite (BT) and synthesized materials (B-BTB, B-DSS, B-BTC and B-BEHA) were characterized via XRD; Powder X-ray diffractometer (PXRD) of Cu-Kα = 1.54 Å in the range of (2θ = 5–70°) with scan rate (2°, 2θ/min), SEM; Scanning electron microscopy (NOVA Nano), EDX; Energy dispersive X-ray spectroscopy for elemental composition and FTIR; Fourier transform infrared spectroscopy, ATR/IRTRACER-100 with resolution rate of (15 scan and 1 cm^-1^) in the range of (4000–400 cm^-1^). In addition, X-ray fluorescence spectrometer (XRF, Bruker S8) was used to determine the chemical composition. TGA study was also investigated for the quantification of organic moieties in the layers of clay by heating in the range of (40–800 °C), inert atmosphere (Helium, 30 mL/min) with heating rate (30 °C/min). Moreover, PETRA-X-ray Fluorescence (XRF) sulfur analyzer (ASTM D-4294) used to analyze the residual amount of sulfur compounds.

## Results and discussion

### Material characterization

#### FT-IR

**Figure 1:**
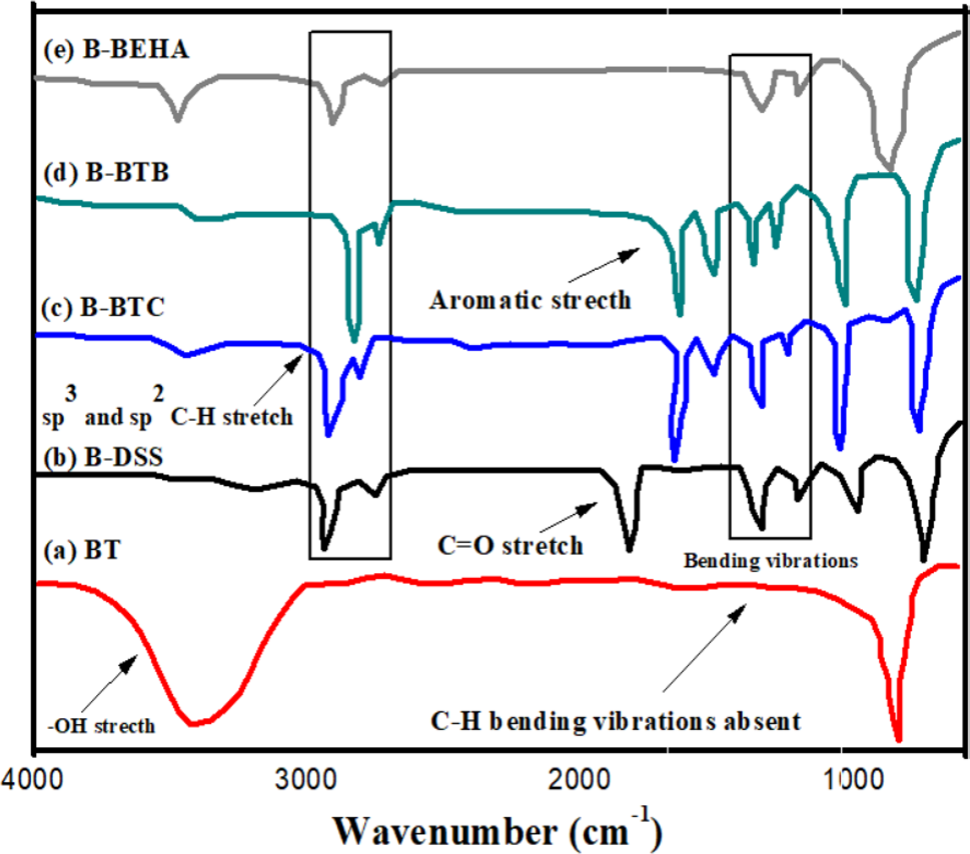
FT-IR spectrum of (**a**) unmodified bentonite and modified bentonite, (**b**) B-DSS, (**c**) B-BTC, (**d**) B-BTB and (**e**) B-BEHA.

In order to affirm the presence of functional groups at the adsorbent surface, FT-IR spectral analysis of both modified and unmodified material (BT) was carried out (range 4000–400 cm^-1^) as shown in Fig. 1. The 3480 cm^-1^ band that appears only in unmodified material due to interlayer water molecules i.e., corresponds to –OH stretching vibrations. The band in the range of 1470–1350 cm^-1^ and 2950–2800 cm^-1^ appears only for modified material is characteristic and corresponds to C–H bending and stretching vibrations, thus absent in unmodified material. The sp^3^ C–H stretch for B-BEHA is (2876 cm^-1^), sp^2^ C–H stretch for B-BEHA (2964 cm^-1^), CH_3_ bending vibration for B-BEHA is (1379 cm^-1^), CH_2_ bending vibration for B-BEHA (1481 cm^-1^). These stretching and bending vibrations are confirms the functional groups of the adsorbent surface that has been modified and are similar to the FT-IR described previously^26,32,33^.

#### XRD and XRF

**Figure 2:**
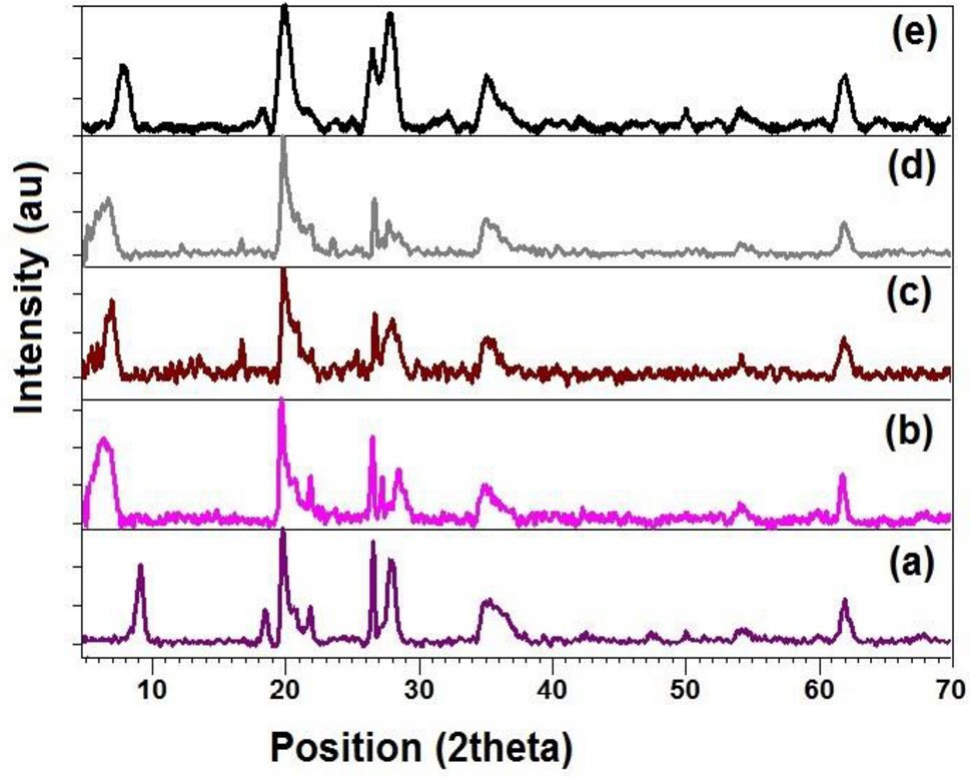
Powder XRD pattern of pristine clay unmodified (**a**), and modified (**b**) B-DSS, (**c**) B-BTC, (**d**) B-BTB, and (**e**) B-BEHA.

Powder X-ray Diffraction studies was carried out to investigate the peak shifting towards lower 2θ value in modified clay that increase the interlayered d-spacing of clay material^26,32,34^. The Fig. 2 shows the pXRD pattern of pristine clay (Fig. 2a) along with the modified clays, most of the peaks in modified pXRD pattern is same as the pristine bentonite clay as confirmed by the Joint Committee on Powder Diffraction Standards card (JCPDS No. 00-003-0015) except one peak that is observed at 2θ = 9.23° with basal d-spacing 0.96 nm in pristine clay. If there is a shifting of this peak observed towards lower 2θ values, it shows the increase in interlayer spacing due to intercalation of organic molecule^26,32,34^. The modified bentonite shows the peak shifting of B-DSS (2θ = 6.44°, 1.37 nm), B-BTC (2θ = 6.97°, 1.26 nm), B-BTB (2θ = 6.79°, 1.30 nm), B-BEHA (2θ = 7.61°, 1.15 nm) (Fig. 2b–e), which are similar to previous reported studies^26,32,34^.

Chemical composition of pristine clay (unmodified) as determined via XRF study was found to be: CaO = 5.01%, Na_2_O = 2.1%, Al_2_O_3_ = 23.9%, MgO = 2.9%, SiO_2_ = 55.1% and K_2_O = 3.48%. This data indicates that; Na, Mg, K and Ca are prime exchangeable cations^24^.

#### SEM

**Figure 3:**
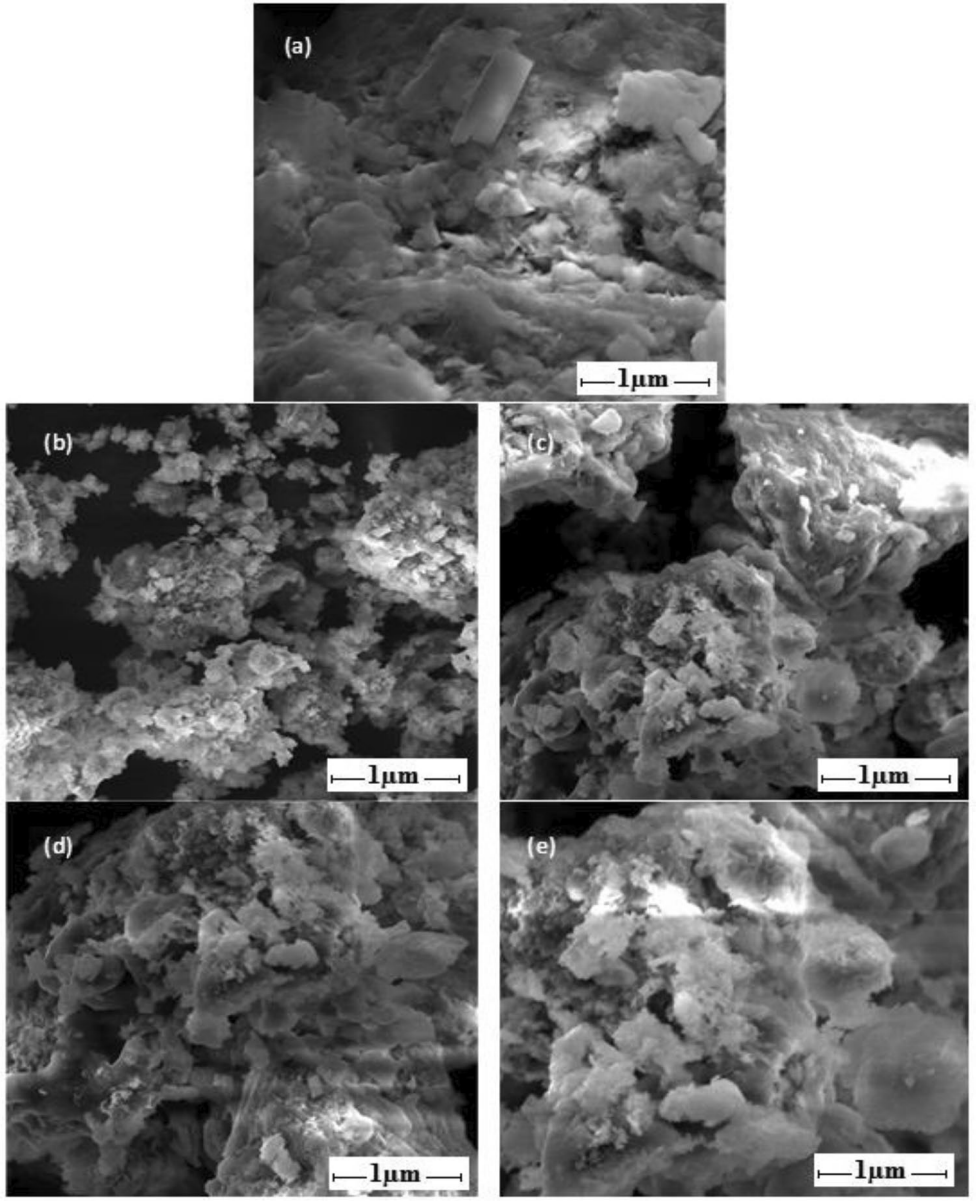
SEM images showing (**a**) unmodified bentonite, and (**b**)–(**e**) modified bentonite (B-BTC, B-BTB, B-DSS and B-BEHA).

The micrographs of unmodified and modified bentonite material were observed via Scanning electron microscopy (SEM Nano NOVA) to confirm the presence of organic moieties into galleries of clay particles and changes in morphology after modification. It seems that the pristine clay exhibits the grasps foliated with massive curved like plates and tightly held as shown in Figure which become foamy, fluffy and more porous after modification as given in Fig. 3^35,36^. Moreover, in modified clay there are bigger porous aggregates that provide more residence to adsorbate and have extra available bonding sites for adsorption of DBT. Additionally, regarding as quantification of elemental analysis in modified clay material via EDX, more carbon content was observed in organoclay than carbon content in unmodified bentonite material because when we loaded the organic molecules (DSS, BTC, BTB and BEHA) into the galleries of clay, the amount of carbon increases in modified materials. The observed carbon content: B-DSS (18.7 wt. %), B-BTC (23.4 wt. %), B-BTB (21.3 wt. %) and B-BEHA (16.9 wt. %) which is absent in unmodified bentonite. The observed components of bentonite clay are present naturally expect carbon content which is observed after modification. The presence of carbon loading indicates the effective synthesis of modified materials (B-DSS, B-BTC, B-BTB and B-BEHA) that results in increase the adsorption capacity of DBT molecules owing to increase in d-spacing of clay galleries after modification as confirmed from XRD.

#### TGA

**Figure 4:**
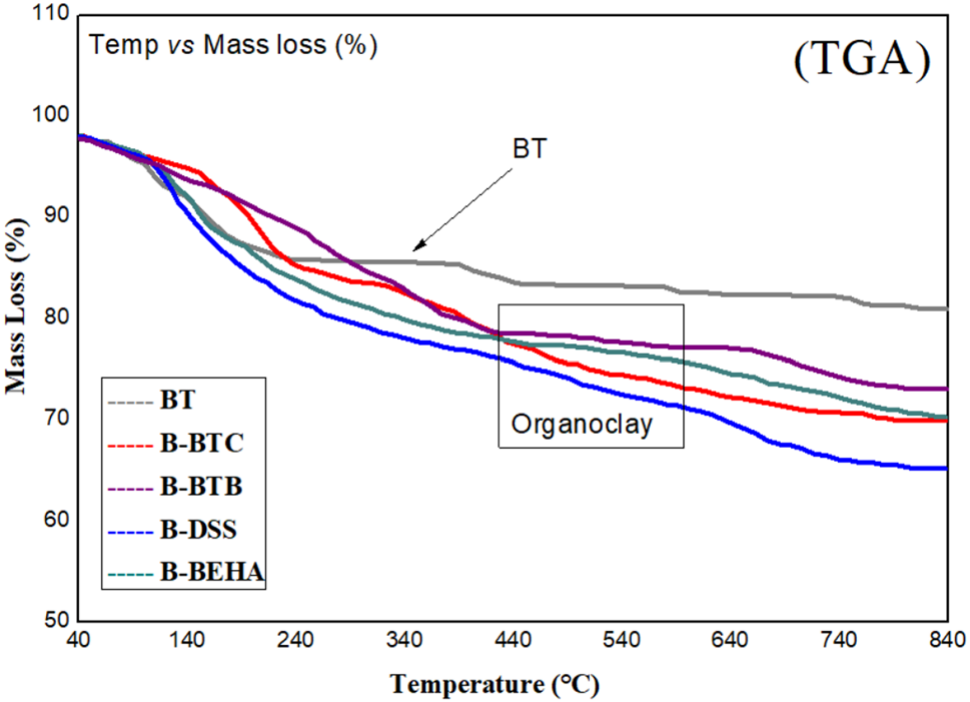
TGA curves of pristine clay BT and modified bentonite material.

The stability of modified and unmodified material was determined by analyzing weight loss over a range of temperature (i.e., 40–840 °C) under inert atmosphere via TGA as given in Fig. 4. Transitions of unmodified material during thermal degradation were at low temperature the surface adsorbed water that volatilize (below 140 °C), at high temperature (450–600 °C) due to –OH group de-hydroxylation of water occurred. The four regions of thermal degradation in modified materials occurred as following; the physically adsorbed gaseous substances and water evolved (below 150 °C), the organic specie (BTC, BTB, DSS and BEHA) decomposed (between 200 and 450 °C), structural water loss caused de-hydroxylation (450–600 °C) and the carbonaceous organic products evolved (between 600 and 700 °C)^24,26^. The increased adsorption capacity of modified materials has successfully been demonstrated by the comparison of modified and unmodified material.

### Optimization of adsorption process

#### Effect of contact time and temperature on DBT adsorption

**Figure 5:**
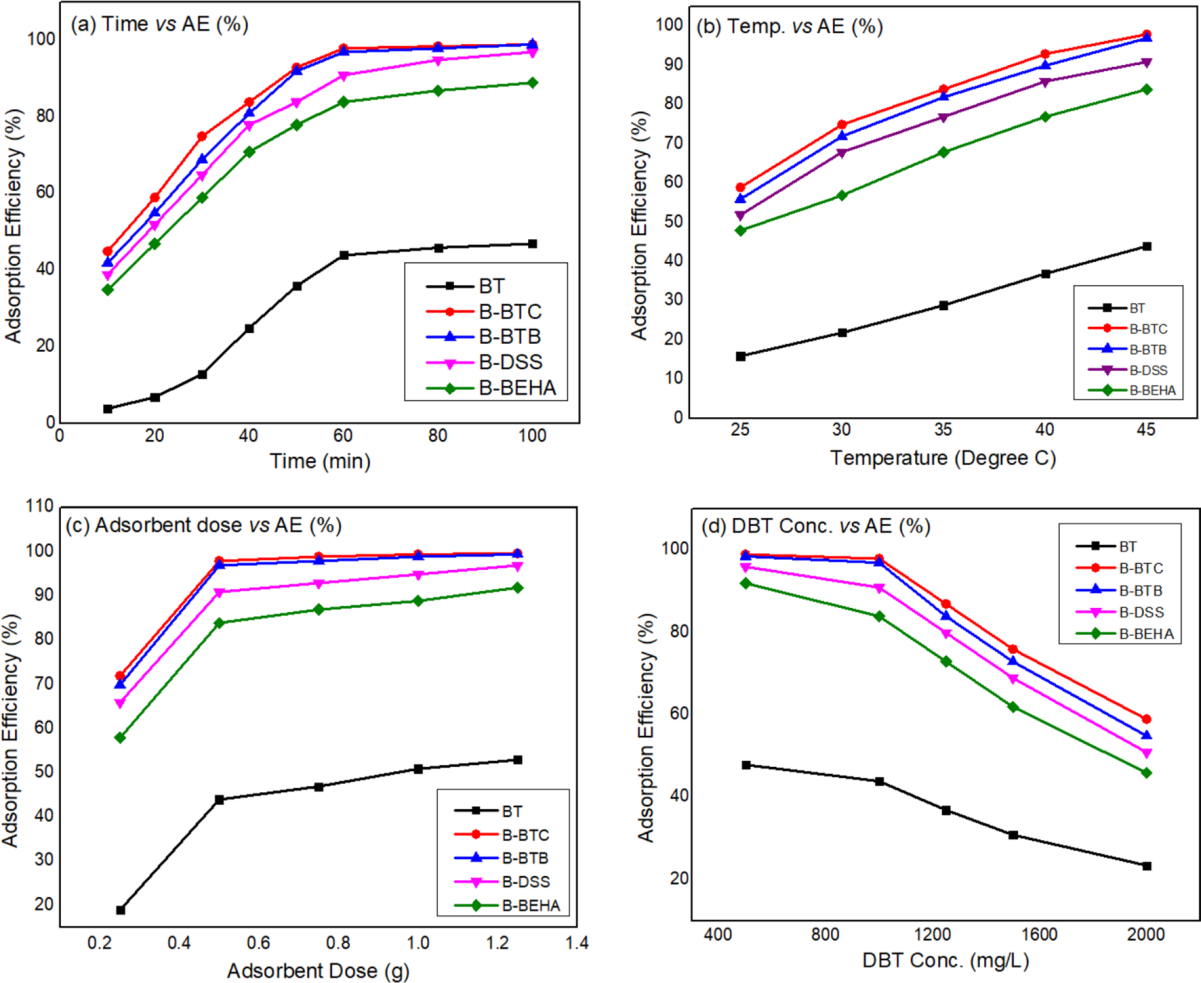
Effect of various parameters: time (**a**), temperature (**b**), adsorbent dose (**c**) and concentration (**d**) on the adsorption DBT.

The effect of contact time on the removal of DBT onto BT, B-BTC, B-DSS, B-BTB and B-BEHA was observed at various time intervals by keeping the adsorbent dose 0.5 g, volume = 30 mL and DBT concentration 1000 mg/L as constant. The adsorption of DBT onto organoclay increases with increase of contact time. At the beginning the adsorption process is fast and gradually slows down in order to attain stability. The maximum adsorption occurs at 60 min. Hence 60 min is marked for higher efficiency of adsorption process as shown in Fig. 5a^17^. Furthermore, to find out the optimum temperature the desulfurization of DBT was carried out by varying temperature in the range of 25–45 $^\circ{\rm C}$ and other parameters were remained constant. The results depict that the adsorption efficiency has direct relation with temperature (Fig. 5b). At higher temperature, Dibenzothiophene (DBT) is more mobile due to reducing the viscosity as well as higher temperature lead the widens of adsorbent pores to some extent and results in decrease the activation energy^19^.

#### Effect of adsorbent dose on adsorption

The effect of adsorbent dosage (organoclay) and unmodified clay on the desulfurization of DBT was investigated by varying the amount of dose (0.25–1.25 g) and the DBT concentration of 1000 mg/L, time = 60 min and volume = 30 mL at 45 $^\circ{\rm C}$ as shown in Fig. 5c. The results declared that adsorption efficiency (%) is directly proportional to adsorbent dose and inversely proportional to adsorption capacity. This decrease in adsorption capacity of DBT with increased adsorbent dose is because of larger number of adsorption sites (organic moieties) as well as mass of adsorbent dose has indirect relation with adsorption capacity (q_e_) as shown in Eq. (2). Hence at lesser adsorbent dosage the adsorption capacity is maximum as observed^19^. The adsorbent (B-BTC) has high percentage of DBT removal than B-BTB, B-DSS and B-BEHA due to presence of two phenyl groups that are more active functional groups and enhances the adsorption proves due to its pi–pi interaction with DBT molecule.

#### Effect of DBT concentration

Adsorption capacity of organoclays is altered by varying the concentration of DBT. Five different concentrations (500–2000 mg/L) were used for the investigation of DBT concentration effects on adsorption of DBT keeping remaining parameters as constant (adsorbent dose = 0.5 g, volume = 30 mL, time = 60 min and at 45 $^\circ{\rm C}$). In general, adsorption capacity increases with increasing concentration of DBT until the availability of the adsorbent. Yet the efficiency is affected, as it limits the available sites (fixed amount of adsorbent) for the DBT molecules (at high levels) but can still work with reduced efficiency. DBT removal and adsorption capacity of organoclay illustrate opposite fashion, which can be elucidated as binding sites of organoclay are fixed. When less concentration of DBT is available the faster will be the adsorption and the percent removal will be high, as higher numbers of binding sites are present on the organoclay^37^. More the presence of binding sites on organoclay, lesser the concentration of the DBT molecules, the most efficient will be the adsorption process as shown in Fig. 5d.

#### Kinetic study

For understanding the mechanism of adsorption process, kinetic study is of prime importance. During adsorption of DBT on to adsorbent (BT, B-BTC, B-DSS, B-BTB and B-BEHA), undergoes various processes from bulk solution onto organoclay surface (adsorbent). For adsorption mechanism, pseudo-first order and pseudo-second order kinetic models were applied. Pseudo-first order is valid for adsorption of adsorbate from aqueous solution (physisorption). The integral form of Pseudo-first order kinetic model is represented as Eq. (3)^38^:

3 $$ \log \left( {q_{e} - qt} \right) = \log \left( {q_{e} } \right) - \frac{{K_{1} }}{2.303}t $$

where q_t_ is amount of DBT adsorbed at time, q_e_ is at equilibrium the amount of DBT adsorbed, K_1_ pseudo-first order constant. Rate constant, intercept and slope were calculated from linear plot (log (q_e_ − q_t_) vs. time).

The pseudo-second order kinetic model is based on “the rate involves forces for sharing or exchanging of electrons between adsorbate and adsorbent (chemisorption)”. Pseudo-second order kinetic model is represented as Eq. (4)^38^:

4 $$ \frac{{dq_{t} }}{dt} = k_{2} (q_{e} - q_{t} )^{2} $$

Rearranging Eq. (4) by integrating within boundary conditions at qt = 0 to t = 0 and qt = qt to t = t, (5):

5 $$ \frac{t}{{q_{t} }} = \frac{1}{{k_{2} q_{e}^{2} }} + \frac{t}{{q_{e} }} $$

where $k_{2}$ pseudo-second order constant, Slope $\left( {\frac{t}{{q_{t} }}} \right)$ and intercept $\left( {\frac{1}{{k_{2} qe^{2} }}} \right)$ were used for calculating $k_{2}$ and q_e_.

**Figure 6:**
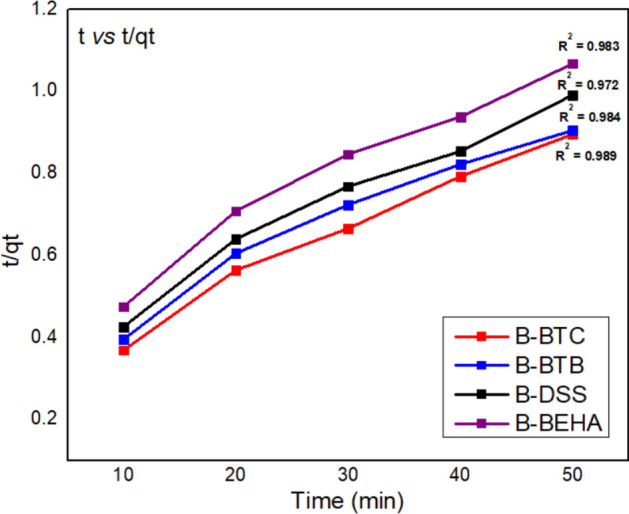
Pseudo second order kinetics for the adsorption of DBT onto organoclay.

The optimized situations for the studies contain 0.5 g of organoclay, 1000 mg/L of DBT concentration with range of time scale as given in Table 1. The regression coefficient (${R}^{2}$) of pseudo-second order is better than pseudo-first order for the organoclay. So, the data indicates the chemisorption mechanism (pseudo second order kinetics) for the adsorption of specific DBT onto modified materials B-DSS, B-BTB, B-BTC and B-BEHA as shown in Fig. 6 and physisorption mechanism (pseudo first order kinetics) followed onto unmodified bentonite material (BT). In addition, the calculated adsorption capacity (q_m_) for pseudo second order kinetics is greater than experimental (q_m_) for the adsorption of DBT via organoclay.

**Table 1:** Kinetics results of Pseudo first order, Pseudo second order and Intraparticle diffusion model for the adsorption of DBT onto BT, B-BTC, B-DSS, B-BTB and B-BEHA.

Adsorbent	Pseudo-first order				Pseudo-second order			Intraparticle diffusion model		
	q _(exp)_ (mg/g)	q_(calc)_ (mg/g)	*K*_1_(min^-1^)	R^2^	q_(calc)_ (mg/g)	K_2_(g/mg min^-1^)	R^2^	K_p_	C	R^2^
BT	26.4	44.98	0.0389	0.852	17	5.36 $\times$ 10^–4^	0.792	4.53	− 13.47	0.917
B-BTC	58.8	67.7	0.0575	0.953	78	5.99 $\times$ 10^–4^	0.983	5.02	15.46	0.881
B-BTB	58.2	74.38	0.0576	0.918	81	4.75 $\times$ 10^–4^	0.972	5.42	11.38	0.899
B-DSS	54.6	60.25	0.0511	0.973	75	5.32 $\times$ 10^–4^	0.984	5.41	9.081	0.936
B-BEHA	50.4	59.06	0.0525	0.960	71	5.16 $\times$ 10^–4^	0.989	5.06	7.587	0.931

To study the mass transfer rate comparison for the adsorption of DBT, the intraparticle diffusion model (*Fickian-diffusion*) model was also applied and it represents as Eq. (6)^38^:

6 $$ {\text{qt}} = K_{p} t^{1/2} + C $$

where C is intercept (determined from q_t_ *vs* t^1/2^ plot) and k_p_ is rate constant (mgg^-1^ min^-1/2^) as values are given in Table 1. This *Fickian-diffusion* model is studied to evaluate the rate controlling step (t = 10–100 min) and the plot is not linear as well as R^2^ (0.881, 0.899, 0.936 and 0.931 for B-BTC, B-BTB, B-DSS and B-BEHA respectively) which is quite lower than pseudo second order kinetics. The obtained findings declared that this model is not fitted well as compared to pseudo second order kinetic model, but it also indicate that the DBT adsorption onto organoclay may also be followed by intraparticle diffusion model.

#### Adsorption isotherm models

Equilibrium adsorption was analyzed by different isotherm models. The amount of adsorbed DBT and its concentration in solution were shown by Langmuir and Freundlich isotherm. Langmuir model shows the adsorption of DBT molecules on to adsorbent surface, happens on homogenous monolayer surface deprived of any interaction with neighboring adsorbed molecules. Langmuir isotherm equation is given as Eq. (7)^24^:

7 $$ \frac{{C_{e} }}{{q_{e} }} = \frac{{C_{e} }}{{q_{m} }} + \frac{1}{{K_{L} q_{m} }} $$

where $K_{L}$ is Langmuir adsorption equilibrium constant associated with free energy and $q_{m}$ is maximum adsorption capacity of organoclay. The adsorption isotherm is plotted by $\frac{{C_{e} }}{{q_{e} }} vs C_{e}$, and data should give straight line, which is appropriate for this model where, slope is $\frac{1}{{q_{m} }}$ and intercept $\frac{1}{{K_{L} q_{m} }}$ . Regarding as Langmuir Isotherm model; there is no interaction among the adsorbed molecules. The distinctiveness of Langmuir isotherm model was expressed via R_L_ parameter (dimensionless constant) that is expressed in Eq. (8):

8 $$ R_{L} = \frac{1}{1 + KL \cdot Co} $$

R_L_ parameter point out the nature of DBT adsorption onto organoclay either; irreversible (R_L_ = 0), unfavorable (R_L_ > 1), favorable (0 < R_L_ < 1) and linear (R_L_ = 1).

Freundlich adsorption isotherm is for multilayer formation that occurs due to heterogeneous adsorption. Freundlich adsorption isotherm equation is given as Eq. (9)^37^:

9 $$ \ln q_{e} = \ln K_{F} + \frac{1}{{n_{F} }}\ln C_{e} $$

where $q_{e}$ is the amount of adsorbate, adsorbed on the surface of adsorbent at equilibrium, $C_{e}$ is equilibrium adsorbate concentration, $K_{F}$ is Freundlich constant and $\frac{1}{{n_{F} }}$ is heterogeneity factor. For favorable adsorption $n_{F}$ is large than 1. The values for slope $\frac{1}{{n_{F} }}$ and intercept $\ln K_{F}$ were obtained from linear plot of ${\text{ ln}}q_{e} vs\ln C_{e}$.

**Figure 7:**
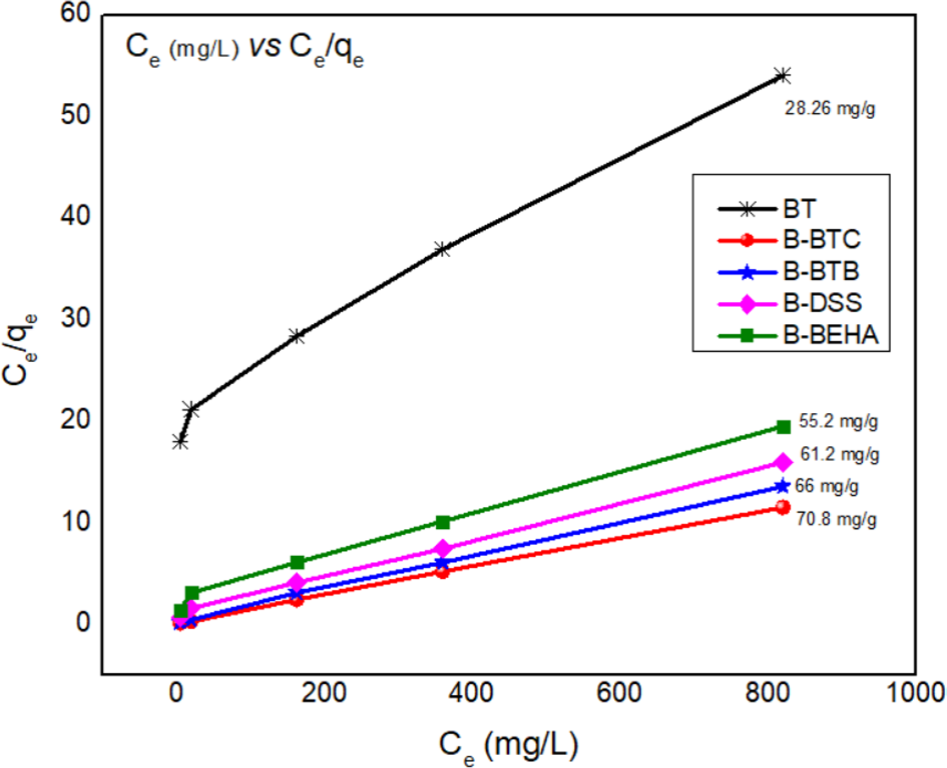
Langmuir Isotherm for the adsorption of DBT onto BT, B-BTC, B-BTB, B-DSS and B-BEHA.

Table 2 and Fig. 7 include the experimental results at optimized conditions (0.5 g adsorbent dose and 1000 mg/L of DBT concentration) of isothermal models, though the regression coefficient (${R}^{2}$) of Langmuir model is best for DBT removal, which indicates the monolayer adsorption of sulfur molecules on the surface of organoclay. Moreover, the calculated value of q_m_ = 33.78 (BT), 70.8 (B-BTC), 66 (B-BTB), 61.2 (B-DSS) and 55.2 (B-BEHA) in (mg/g) by using Langmuir model is very close to the experimental observed q_m_ = 28.26 (BT), 71.43 (B-BTC), 66.66 (B-BTB), 62.50 (B-DSS) and 57.14 (B-BEHA) mg/g which clearly indicated the monolayer adsorption phenomenon. The R_L_ value is lesser than 1 which indicating the favorability of DBT adsorption onto both unmodified and organoclay. Moreover, the order of adsorptive desulfurization is B-BTC > B-BTB > B-DSS > B-BEHA > BT.

**Table 2:** Isotherm studies for the adsorption of DBT onto modified bentonite.

Adsorbent	Langmuir					Freundlich			Temkin Model	
	q_m_ (mg/g)	Exp.q_m_ (mg/g)	K_L_(L/mg)	R^2^	RL	nF	Kf (mg/g)	R^2^	B	R^2^
BT	33.78	28.26	0.004	0.97	0.193	2.62	1.98	0.769	7.87	0.792
B-BTC	71.43	70.8	0.104	0.99	0.009	6.71	28.8	0.753	7.19	0.8166
B-BTB	66.66	66.00	0.129	0.99	0.007	6.72	26.9	0.724	6.89	0.7724
B-DSS	62.50	61.20	0.072	0.99	0.013	5.23	18.9	0.782	8.36	0.8175
B-BEHA	57.14	55.2	0.040	0.99	0.024	4.72	14.4	0.788	8.54	0.8146

To examine the adsorbate-adsorbent interaction for the adsorption of DBT onto unmodified and modified bentonite, we further applied a Temkin model. The general representing of Temkin model is given in Eq. (10)^19^:

10 $$ q_{e} = BlnC_{e} + BlnA $$

where A (Temkin constant (L/g) that is related to adsorbate–adsorbate interaction), B (Heat of adsorption in J/mol) and q_e_ (equilibrium adsorbed amount (mg/g)). The heat of adsorption and regression coefficient (R^2^) is given in Table 2 which indicates that the Temkin model is not well fitted as compared to Langmuir isotherm model.

#### Thermodynamic studies of DBT adsorption

To understand the effect of different temperatures for the removal of DBT from fuel oil via organoclay, various thermodynamic paramters i.e., standard entropy, standard enthalpy and standard Gibbs Free energy has been thoroughly studied. The above procedure was conducted with 30 mL of (1000 mg/L) initail (DBT) solution at various temperatures (298.5, 303.5, 318.5, and 333.5 K) along with 0.5 g (modified clay) for an hour. Using following Eq. (11) Gibbs Free energy was calculated^19^:

11 $$ \Delta G^\circ = - RT\ln K_{c} $$

Moreover, Standard entropy was calculated using Vant’s Hoff Eq. (12) by plotting InK_c_ *vs* 1/T and standard enthalpy was calculated using Eq. (13)^39^:

12 $$ lnK_{c} = - \frac{\Delta H^\circ }{{RT}} + \frac{\Delta S^\circ }{R} $$

13 $$ \Delta G^\circ = \Delta H^\circ - T\Delta S^\circ $$

where K_c_ = Organoclay retention ability to hold the (DBT) which is calculated through Eq. (14):

14 $$ K_{c} = \frac{{q_{e} }}{{C_{e} }} $$

Here C_e_ is the adsorbed Organoclay equilibrium concentration while q_e_ is the (DBT) equilibrium concentration.

The van der wall forces exist between organoclay (B-BTC, B-DSS, B-BTB and B-BEHA) and DBT molecules were reduced by triggers the weak interaction at low temperature and at high temperature optimum adsorption was observed, thus it gives neagtive values of $\Delta G^\circ$. However , positive value of enthalpy was justified the endothermic nature of adsorption. Besides this during the adsorption of (DBT), the positive values of $\Delta S^\circ$ indicates the irregularity in randomness onto synthesized oragnoclay as given in Table 3.

**Table 3:** Thermodynamic parameters for the adsorption of Dibenzothiphene (DBT) on modified clay.

Adsorbents	$\Delta G^\circ (KJ{mol}^{-1})$				$\Delta H^\circ$ $\left(KJ{mol}^{-1}\right)$	$\Delta S^\circ$ $(KJ{mol}^{-1}{K}^{-1})$
	T = 298.5 K	T = 303.5 K	T = 318.5 K	T = 333.5 K		
BT	11.09	10.29	9.82	8.469	0.557	0.002
B-BTC	− 21.87	− 32.37	− 83.64	− 106.83	4.393	5.114
B-BTC	− 20.22	− 29.88	− 77.58	− 94.59	4.308	5.028
B-DSS	− 18.15	− 26.89	− 68.25	− 89.78	3.985	4.874
B-BEHA	− 12.42	− 20.17	− 56.28	− 74.62	2.545	3.657

#### Adsorptive desulfurization (ADS) of real fuel oil

The adsorptive desulfurization of commercially available fuel samples (Kerosene and Diesel) was also investigated via modified bentonite material (B-BTC) under optimized conditions (time = 60 min, adsorbent = 0.5 g, volume = 30 mL and temperature = 45 $^\circ {\text{C}}$) and before desulfurization the total sulfur content in kerosene and diesel oil was found to be 2848 mg/L and 4468 mg/L respectively. The concentration is too high to be removed under the optimized conditions, therefore the dilution of the sample was carried out with iso-octane to get the sulfur content in the range of 1200 mg/L. To quantify the amount of sulfur components PETRA X-Ray Fluorescence Spectrophotometer (XRF) (ppm, ASTM D-4294) was used. Moreover, other fuel properties such as specific gravity, water content and distillation were also conducted via Hydrometer (g/mL @ 15.6 $^\circ {\text{C}}$, ASTM D-1298), Water content tester (China PT-D4006-8929A) (vol. %, ASTM D-4006) and Distillation tester (PMD 110, PAC) (ASTM D-86). The findings declared that 96.76% and 95.83% removal efficiency was achieved for kerosene and diesel oil respectively and the other fuel characteristics before and after ADS are given in Table 4. Moreover, the unmodified bentonite was also tested for the desulfurization of fuel oil but due to lower interlayer spacing of clay the results was not efficient as compared to modified bentonite material. Upon modification the increase in *d*-spacing and development of interesting morphology (porous and fluffy) results in the increase in adsorption capacity.

**Table 4:** ADS of kerosene and diesel oil onto B-BTC.

Tests		Method No	Kerosene		Diesel oil	
			Before ADS	After ADS	Before ADS	After ADS
Specific Gravity (g/mL @ 15.6 $^\circ {\text{C}}$)		ASTM D-1298	0.834	0.830	0.882	0.875
Total sulfur by Petra X-Ray, wt. % (ppm)		ASTM D-4294	1200	40	1200	50
Water content by distillation (vol. %)		ASTM D-4006	Nil	Nil	Nil	Nil
Distillation $(^\circ {\text{C}})$	50%	ASTM D-86	240	240	280	279
	90%		301	299	346	342

#### Compariosn with other reported methods

Due to limited available data for the DBT desulfurization via organo-clay based modified materials, we can not make comparison for the adsorption efficiency effectively. Moreover, we have compared adsorpton capacity (mg/g) for the DBT removal with other modified adsorbents as given in Table 5. It can be seen that the proposed method shows better adsorption capacity than the reported methods.

**Table 5:** Comparison of adsorption capacity (mg/g) of various adsorbents for desulfurization.

Adsorbent	Adsorption Capacity (mg/g)	Initial Sulfur Conc. (mg/L)	References
Granulated activatd carbon	3.52	324	^40^
Copper impregnated activated carbon	3.34	333	^40^
Iron impregnated activated carbon	3.59	320	^40^
Nickel impregnated activated carbon	3.52	324	^40^
Aluminium Oxide	2.79	360.5	^41^
Bentonite	2.29	271.2	^9^
Activated clay	4.01	99.5	^9^
Kaolinite	1.73	327	^9^
Carbon nanotubes	21.50	250	^42^
AC_TD_	8.60	150	^43^
Carbon silica nanocomposite via Cu-modified	13.95	960	^44^
Carbon aerogels	15.10	250	^45^
Magnetic mesoporous carbon	62	1000	^46^
B-BTC	70.8	1000	Present study
B-BTB	66.00	1000	Present study
B-DSS	61.20	1000	Present study
B-BEHA	55.2	1000	Present study

## Conclusions

The cost-effective material has been developed via benzyl tri-n-butyl ammonium bromide (BTB), Dioctyl sodium sulfosuccinate (DSS), benzethonium chloride (BTC) and Bis (2-ethylhexyl) amine (BEHA) based modified bentonite materials (B-BTC, B-BTB, B-DSS and B-BEHA) for studying the adsorption of model fuel oil and commercially available real fuels (Kerosene and Diesel oil). XRD and SEM depicts the increase in interlayer spacing and more porous/fluffy structure upon modification. The result of present study shows promising achievement (adsorption capacity 70.8 (B-BTC), 66 (B-BTB), 61.2 (B-DSS) and 55.2 (B-BEHA) and adsorption efficiency > 90% for B-BTC, B-BTB and B-DSS and > 80% for B-BEHA) which are greater than unmodified material (28.26 mg/g) under optimized operating conditions for prepared model oil. Additionally, the findings declare that 96.76% and 95.83% removal efficiency was achieved for kerosene and diesel oil, respectively, at optimized conditions and fuel properties follow ASTM specifications. The optimization study shows direct relation of adsorption efficiency with time, temperature and adsorbent amount and is indirectly related to DBT concentration. Adsorption kinetics study follows pseudo-second-order kinetic model (regression coefficient R^2^ = 0.98) which shows chemisorption behavior of DBT adsorption onto modified materials and followed pseudo first order (physisorption) onto unmodified material. However, present data fits very well with isothermal (Langmuir Model where 0 < R_L_ < 1 shows favorable adsorption and R^2^ = 0.99) and thermodynamic studies (endothermic and spontaneity in system). Thus, the whole analytical study confirms the prominence of developed organoclay material for better adsorptive desulfurization.
